# A Single Base Insertion in *F9* Causing Hemophilia B in a Family of Newfoundland–Parti Standard Poodle Hybrid Dogs

**DOI:** 10.3390/genes12101491

**Published:** 2021-09-24

**Authors:** Henrike Kuder, Liubov Sandzhieva-Vuzzo, Alexandra Kehl, Jonathan M. Rappaport, Elisabeth Müller, Urs Giger

**Affiliations:** 1 Laboklin GmbH & Co. KG (Labogen), Steubenstraße 4, D-97688 Bad Kissingen, Germany; henrike.kuder@uzh.ch (H.K.); kehl@laboklin.com (A.K.); mueller@laboklin.com (E.M.); 2 Vetsuisse Faculty, University of Zürich, Winterthurerstrasse 260, CH-8057 Zürich, Switzerland; 3 Advanced Veterinary Care Center, 8920 W. State Road 84, Davie, FL 33324, USA; liubovsandzhieva@gmail.com (L.S.-V.); jrappaport@advetcc.com (J.M.R.); 4 Section of Medical Genetics (PennGen), School of Veterinary Medicine, University of Pennsylvania, 3900 Delancey Street, Philadelphia, PA 19104, USA

**Keywords:** bleeding disorder, coagulopathy, factor IX, frameshift mutation, canine, intrinsic pathway, X-linked recessive, partial thromboplastin time

## Abstract

Hemophilia B is an x-linked recessive hereditary coagulopathy that has been reported in various species. We describe a male Newfoundland–Parti Standard Poodle hybrid puppy and its family with hemophilia B from clinical manifestations to the molecular genetic defect. The index case presented for dyspnea was found to have a mediastinal hematoma, while surgical removal and transfusion support brought some relief, progressive hematoma formations led to humane euthanasia. Sequencing the *F9* exons revealed a single nucleotide insertion resulting in a frameshift in the last exon (NM_001003323.2:c.821_822insA), predicted to result in a premature stop codon (NP_001003323.1:p.Asn274LysfsTer23) with a loss of 178 of 459 amino acids. The unexpected high residual plasma factor IX activity (3% to 11% of control) was likely erroneous, but no further studies were performed. Both the purebred Newfoundland dam and her sister were heterozygous for the insertion. Five additional male offspring developed severe hemorrhage and were hemizygous for the *F9* variant and/or had a prolonged aPTT. In contrast, other male littermates had normal aPTTs and no evidence of bleeding. While they are related to a common Newfoundland granddam, the prevalence of the pathogenic variant in the Newfoundland breed is currently unknown. These clinical to molecular genetic studies illustrate that precision medicine is achievable in clinical companion animal practice.

## 1. Introduction

Hemophilia A and B are X-linked recessively inherited coagulopathies that may cause severe bleeding in humans and domestic animals. The conditions are caused by mutations in the *F8* or *F9* gene, leading to reduced plasma activity of coagulation factor (F) VIII or IX, respectively [1,2,3,4,5,6]. Several *F8* and *F9* gene variants have been reported in bleeding dogs of various breeds [7,8,9,10,11,12,13] and are listed in the Online Mendelian Inheritance of Animals database (OMIA [14], Hemophilia A 000437-9615, and Hemophilia B 000438-9615).

This clinical and molecular genetic study describes a male Newfoundland–Parti Standard Poodle hybrid (NewfyPoo) puppy with hemophilia B and severe clinical bleeding due to a novel *F9* frameshift variant, and the genotypic and phenotypic assessment of its family members.

## 2. Materials and Methods

### 2.1. Dogs and Samples

The index case, a privately owned NewfyPoo, is designated Case #1. He and his relatives were assessed clinically and diagnostically. Coagulation factor analyses were performed in fresh frozen citrated plasma at Comparative Coagulation Laboratory, Cornell University, Ithaca, NY, USA while molecular genetic studies were done at Labogen (Laboklin GmbH & Co. KG, Bad Kissingen, Germany). To determine the molecular genetic defect in the index case, ethylenediamine tetraacetic acid (EDTA)-anticoagulated blood samples left-over from routine hematology testing were used. Genotyping by buccal swabs was then offered to the breeder and owners of related dogs for informed medical management and breeding plans. In addition, archived DNA samples from Newfoundland dogs (including Landseers) and Standard Poodles sent for routine diagnostic testing at Labogen were examined for the presence of the novel *F9* variant. The use of left-over samples at Labogen was approved by the governmental animal care and use committee in Bavaria, Germany.

### 2.2. Molecular Genetic Analysis

Genomic DNA was isolated from blood and buccal swab samples with GenElute™ Blood Genomic DNA Kit (Sigma Aldrich/ Merck, Darmstadt, Germany) and the MagNA Pure 96 system using DNA Tissue Lysis Buffer and viral NA Small kit (Roche, Basel, Switzerland), according to manufacturers’ instructions. Based upon the published reference canine *F9* genome DNA sequence (CanFam3.1, NC_006621.3 [15]), primers were designed in adjacent intronic sequences to amplify all eight canine *F9* exons including exon-intron boundaries and splice sites (Supplemental Table S1). Primers were synthesized by Eurofins Genomics GmbH (Ebersberg, Germany). DNA fragments were amplified by polymerase chain reaction (PCR) utilizing established protocols with a premixed FastStart Mastermix (Roche), and an automated thermal cycler (Bioer Technology Co., Ltd., Hangzhou, China).

Sanger sequencing was performed with an ABI Genetic Analyzer 3130 (Applied Bioscience, Thermo Fisher Scientific, Waltham, MA, USA), and the amplified products were analyzed with a BigDye^TM^ Terminator v1.1 Cycle Sequencing Kit (Thermo Fisher Scientific). Electropherograms were assessed by SeqScanner (Applied Biosystems) [16], and DNA sequences were aligned to published cDNA sequences for canine *F9* (NC_006621.3 (CanFam3.1), NC_049780.1 (UNSW_CanFamBas_1.0), NC_049299.1 (UMICH_Zoey_3.1), NC_049260.1 (UU_Cfam_GSD_1.0), NC_006621.4 (Dog10K_Boxer_Tasha), NC_051843.1 (ROS_Cfam1.0) [15] using an open-source multi-alignment tool from the National Center for Biotechnology Information (Nucleotide Blast) [17]. The *F9* DNA sequences of Case #1 were compared to an unrelated healthy Newfoundland dog sequenced from an archived DNA sample. The Dog Biomedical Variant Database Consortium (DBVDC), which contains genomes from 582 dogs representing 126 breeds, including two Newfoundland dogs, one Landseer, and two Standard Poodles, was screened by BCF-tools view for the VCF file DBVDC [18]. To determine the prevalence of the putative pathogenic variant in the Newfoundland and Poodle breeds, DNA samples from additional 75 Newfoundland dogs and 75 Poodles were genotyped. A web-based DNA translation tool from ExPASy (SIB Bioinformatics Resource Portal) [19] was applied to predict the FIX amino acid sequence.

### 2.3. Genotyping

For genotype screening of DNA samples, a variant-specific TaqMan^®^ SNP Genotyping Assay (Thermo Fisher Scientific) was designed utilizing the published genomic DNA reference sequence of canine *F9* (CanFam3.1, NC_006621.3 [15]), (Supplemental Table S1). To detect the mutant allele sequence, the probe was labeled with FAM^TM^ dye, the wild-type allele probe was labeled with VIC^TM^ dye (Thermo Fisher Scientific). A Rotor-Gene 6000 (Corbett) was used for amplification by standard real-time PCR protocol as well as allelic discrimination.

### 2.4. Family Study

The breeder of Case #1 was contacted and clinical, laboratory test results, and breeding information related to the kennel were obtained to determine the potential extent of the disease in the family and prepare a pedigree for the index case. Based on the diagnosis of hemophilia B in the index case, the dam (purebred Newfoundland dog) and sire (purebred Standard Parti Poodle) were tested by the breeder for published *F9* variants by Wisdom Panel (Portland, OR, USA) [20] and Vetnostics Laboratories (Hamilton Township, NJ, USA) [21] prior to our involvement. After being contacted by us, the breeder then tested all related male offspring with an activated partial thromboplastin time (aPTT) at their primary care clinics. Once the pathogenic *F9* variant was discovered in Case #1, genotyping with buccal swabs from the dogs related to the index case was offered to the breeder free of charge. Breeders and owners of additional maternal ancestors for the index case were contacted to examine the genotypes of their dogs, but these animals were either deceased or not made available.

## 3. Results

### 3.1. Case Report

A three-month-old male intact NewfyPoo dog (index case, Case #1) was presented with lethargy, poor appetite, and unlocalizable pain to the Advanced Veterinary Care Center in Davie, FL, USA (Day 1). Other than mild bleeding from the mouth after losing a deciduous tooth the day prior to presentation, no evidence of abnormal bleeding was previously observed in this individual or any littermates. Values of a routine complete blood cell count, serum chemistry screen, and prothrombin time (PT) of Case #1 were within the reference ranges for puppies, but the activated partial thromboplastin time (aPTT) was markedly prolonged (240 s; reference range, 72–102 s; Table 1).

**Figure 1:**
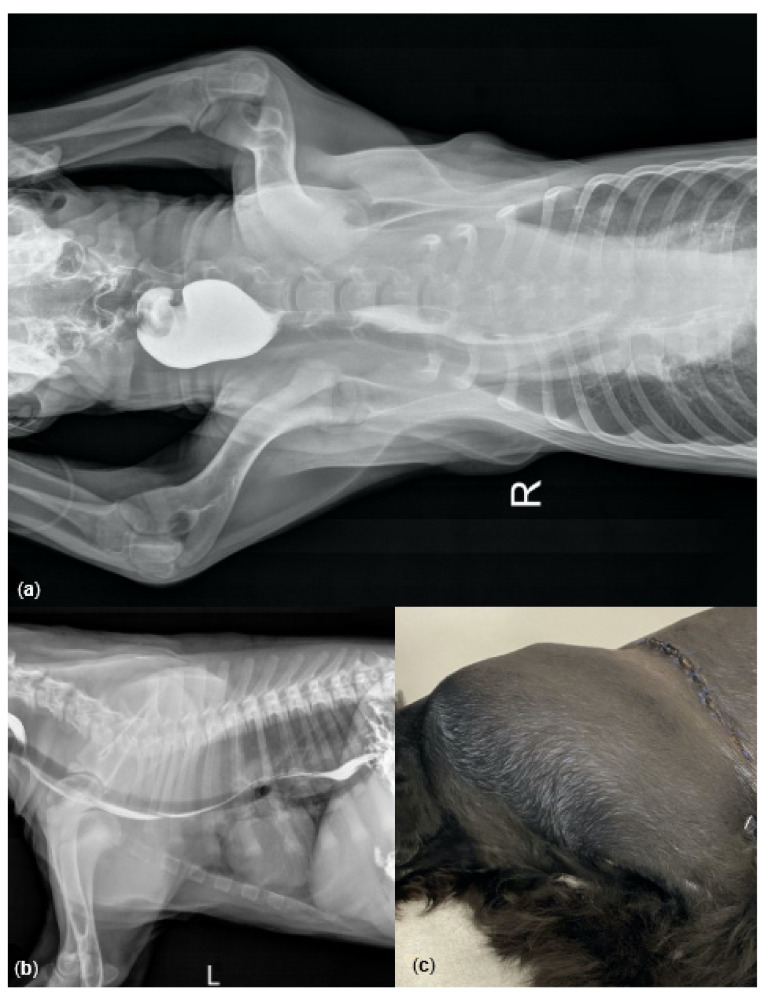
Clinical features showing chest radiographs and hematomas in a Newfoundland–Parti Standard Poodle hybrid puppy with hemophilia B: Case #1. Radiographic views of ventrodorsal (**a**) and lateral (**b**) thorax after a bolus of barium suspension showing tracheal and esophageal displacement at the time of admission (Day 1). Photograph of left chest wall post-surgery on Day 10 showing a large subcutaneous hematoma (**c**).

Thoracic radiographs showed a soft tissue mass in the mediastinum causing ventral displacement of the esophagus and trachea (Figure 1a,b). The puppy was hospitalized for diagnostic evaluation and supportive care, which included blood type and crossmatched transfusions. The next morning (Day 2), the puppy had to be intubated due to labored breathing. In addition to the mediastinal mass, pleural effusion was detected by ultrasound. Based on the isolated prolongation of the aPTT, a hereditary coagulopathy was suspected, and citrated plasma for coagulation factor analyses was sent to Comparative Coagulation Laboratory, Cornell University, Ithaca, NY, USA. The results received on Day 7 revealed a low plasma FIX activity consistent with hemophilia B (Table 2).

Extubation was attempted on Day 3, but the puppy became very dyspneic. Thoracocentesis did not improve dyspnea, and a thoracotomy to drain the chest and stop bleeding was performed under general anesthesia with assisted ventilation. Purpura covering the entire mediastinum was observed, but there was no active bleeding. The large mediastinal mass causing airway compression was removed. Based on gross appearance and histology, the mass was identified as a hematoma.

Fresh frozen plasma (FFP; 20 mL/kg), packed red blood cells (pRBCs; 16 mL/kg), crystalloid, and canine albumin were administered intravenously on Day 4. After FFP administration, the aPTT was only slightly prolonged (126 s; Table 1). The puppy appeared to respond well to post-surgical care and was discharged on Day 7.

On Day 10 (eight days after surgery), the puppy was presented for lethargy, a 10 × 20 cm subcutaneous hematoma over the left shoulder (Figure 1c), and right forelimb lameness. During the examination no signs of dyspnea were observed, and the thoracotomy skin incision seemed to be healing well (Figure 1c). The aPTT was again severely prolonged, and a citrated plasma sample was submitted for confirmatory coagulation factor analyses which revealed a plasma FIX activity of 3% (previously 11%), without evidence of neutralizing anti-FIX antibodies (also called inhibitors; Table 3). Diagnosis of hemophilia B, severe recurring hemorrhage, and an expected poor prognosis in large breed dogs [3] led the owner to elect humane euthanasia. A necropsy was not performed.

### 3.2. Molecular Genetic Analysis

**Figure 2:**
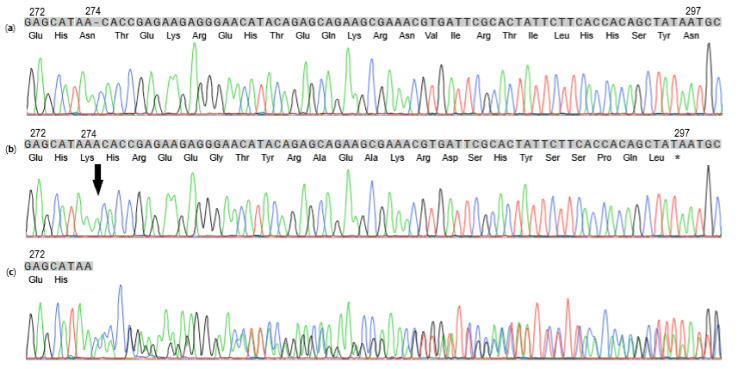
Sanger sequence data for the region of *F9* surrounding the single base insertion variant for a wild-type Newfoundland dog (**a**), a Newfoundland–Parti Standard Poodle with hemophilia B (Case #1) (**b**), and the dam for Case #1 (**c**). The *F9* sequence for the 5´ region of exon 8 and the corresponding amino acid sequence are shown. Sequence for the hemizygous index case with the single adenine base insertion NM_001003323.2:c.821_822insA (arrow) results in a reading frameshift. The first predicted premature stop codon NP_001003323.1:p.Asn274LysfsTer23 is marked by an asterisk. Sequence for the dam of the index case shows heterozygosity for the insertion. The top lines show the DNA codons in grey boxes, with the predicted amino acid sequences below, starting with amino acid 272 in the canine amino acid sequence (NP_001003323.1), which is concordant to amino acid 281 in the human amino acid sequence (NP_000124.1) of *F9*.

**Figure 3:**
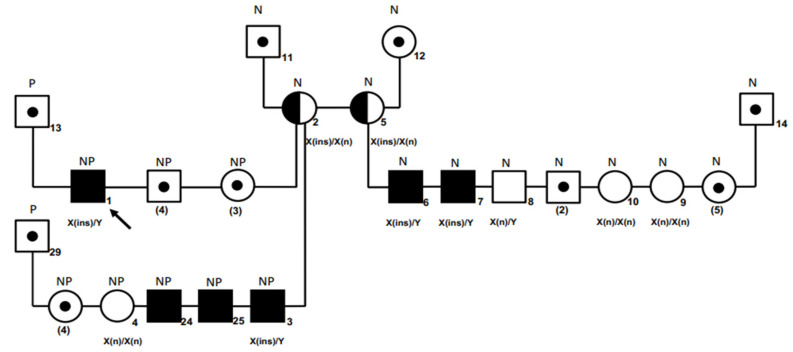
Pedigree of a hemophiliac Newfoundland–Parti Standard Poodle family, with results of genotyping and aPTT results. Squares and circles represent males and females, respectively. Filled symbols indicate clinically bleeding male dogs hemizygous for the *F9* variant and/or having a prolonged aPTT. All affected animals displayed abnormal bleeding. Half-filled symbols indicate females who are carrier of the *F9* variant. The black arrow refers to the index case (Case #1). Symbols with a dot but with numbers in brackets indicate number of dogs that were not available for genotyping. The six male offspring in this category had normal aPTTs (squares with dot). Genotyping results are shown below each square or circle (X and Y represent the X- and Y-chromosomes; (n), wild-type; (ins), insertion (NM_001003323.2:c.821_822insA [15]). Abbreviations: N, purebred Newfoundland dog; NP, Newfoundland–Parti Standard Poodle: P, Standard Poodle.

Exonic and adjacent intronic *F9* gene sequence for Case #1, the published canine reference genome sequence (CanFam3.1, NC_006621.3), recent canine reference genome sequences (NC_051843.1, NC_049260.1, NC_006621.4) [15] and the genomic sequence of a healthy unrelated Newfoundland dog were identical except for a single non-synonymous variant: a single adenine base insertion in exon 8 of the *F9* gene, ChrX:g.109,531,586_109,531,587insA (CanFam3.1, NC_006621.3) [15] in Case #1. The NM_001003323.2:c.821_822insA single base insertion results in a frameshift that changes the wild type amino acid sequence starting at position NP_001003323.1:p.Asn274Lys [15]. After 23 amino acids, there is a predicted premature stop codon (NP_001003323.1:p.Asn274LysfsTer23) (Figure 2 and Figure 3) [15,19,23]. This stop codon at position 297 is followed by additional premature stop codons at NP_001003323.1:p.Asn301* and NP_001003323.1:p.Lys302*.

### 3.3. Family and Breed Studies 

Using *F9* primer pair 8 (Supplemental Table S1) for exon 8, DNA samples from ten related and one unrelated Newfoundland dog were sequenced. The unrelated Newfoundland dog’s DNA had no sequence variations compared to published wild-type sequences. Moreover, screening by TaqMan^®^ SNP Genotyping Assay of archived samples from 75 Newfoundland dogs and 75 Poodles did not reveal other carriers or affected animals (all homozygous wild-type).

Within the family, the dam (#2) of the index case (#1) and her sister (#5) were both heterozygous for the *F9* exon 8 insertion, and three of their male puppies (#3, #6, #7) were hemizygous for the insertion. Male #8, female #4 (a littermate of the index case), and two female offspring (#9, #10) of #5 and a Parti-Standard Poodle were homozygous wild-type (Figure 3). 

The breeder had all male offspring of the three litters tested by aPTT. Three NewfyPoo hybrids and two Newfoundland dogs were found to have a prolonged aPTT (#3, #6, #7, #24, #25), while six other males had normal aPTT values. Three males with a prolonged aPTT were hemizygous for the *F9* variant (#3, #6, #7; Figure 3). Shortly thereafter, they developed intermittent lameness with joint swelling (hemarthrosis). One of them also developed severe internal hemorrhage and was euthanized. Another bled after vaccination at eight weeks of age and was euthanized. Initially, unaware of the bleeding disorder, the breeder had sold all puppies to be neutered and not to be bred. The breeder of the common granddam of the hemizygous males was contacted and was unaware of a bleeding disorder in any of the dogs or their offspring until notified of the index case by the breeder and authors. The granddam was not available for testing (Figure 3). The great-granddam was not bred after the litter producing the granddam of the index case and had since passed away.

## 4. Discussion

This is the first report of hemophilia B in Newfoundland dogs which has also affected Newfoundland–Standard Poodle hybrids. The pathogenic mutation is a single base insertion in exon 8 of *F9* (NM_001003323.2:c.821_822insA) on the X-chromosome that causes a frameshift encoding a premature stop codon after 23 amino acids (NP_001003323.1:p.Asn274LysfsTer23) [15,19,23]. The variant is in the last exon of *F9*, which encodes the major portion of the FIX catalytic domain [24]. The insertion results in the loss of 178 of 459 amino acids, including the protease active site serine residue. While a circulating non-functional truncated form of FIX may be present in the plasma of affected animals, no samples were available, and no further protein and expression studies were performed. An early stop codon leads typically to premature mRNA decay when it is located at least 50 nucleotides upstream of the exon-exon junction. As the identified *F9* variant in the Newfoundland dogs is located in the last exon, premature mRNA decay appears unlikely [25].

The amino acid asparagine at position 274 in canine FIX protein, corresponding to residue 283 in human FIX, is highly conserved among mammalian species (Supplemental Figure S1 [26]). While no nonsense variants at this position have been reported in any human patient with hemophilia B, a missense variant (p.Asn283Asp) at this location was described in one human patient [27]. The European Association for Haemophilia and Allied Disorders (EAHAD) Coagulation Factor IX Gene (*F9*) Variant Database [28,29] (accessed September 20, 2021) currently lists 1244 variants in *F9* including insertions and deletions. Slightly more than half (56.9%) are located in the catalytic domain (exons 7 and 8), and the rest involve the remainder of the protein from signal-/pro-peptide to the activation peptide. Of the 206 frameshift variants listed in the human database, 66 insertions or deletions causing frameshifts reside in exon 8 [28,29].

Only a few naturally occurring *F9* variants have been reported in animal species (Online Mendelian Inheritance in Animals [OMIA] [14], accessed August 21, 2021), and most of these have been reported in the domestic dog (Table 3). A missense *F9* variant in a research colony descendent from an affected Cairn Terrier reported in 1989 represents the very first discovered pathogenic gene variant in the dog [7]. Thereafter, complete and large *F9* gene deletions, insertions, nonsense, and missense variants were found in different canine breeds [7,8,9,10,11,12,13]. In contrast to a single base insertion as in hemophiliac Newfoundland dogs in this report, truncations of the FIX protein due to a large 5 kb insertion or five nucleotide deletion in the terminal exon 8 of *F9* were found in the Airedale Terrier [10] and Lhasa Apso [8], respectively (Table 3).

Consistent with the predicted complete loss of FIX function, the aPTTs of the index case and related affected Newfoundland and hybrid dogs were severely prolonged, and all affected dogs had a severe bleeding tendency. However, the plasma FIX activities of the index case were not as expected < 1%, but 11% to 3% on two separate measurements. The first plasma sample was collected prior to blood transfusion, while the second sample was at least four days after the last plasma transfusion. The half-life of human FIX is 18- 24 h [1], suggesting that the second plasma FIX activity value of 3% from the index case may reflect the prior transfusion. Plasma factor activities of the intrinsic coagulation cascade are assessed with aPTT assays and are reported as percentages of the activity in normal human plasma, which by definition has a species-specific 100% activity of plasma pooled from several healthy dogs (100% control). It is possible that there was activation of FVII or other factors in the common pathway leading to fibrin formation and an erroneous FIX activity value of 11%.

A phenotype to genotype comparison of the few other published canine cases of hemophilia B shows a good correlation. Plasma aPTT and FIX activities were markedly reduced to <5%, and for those expected to be cross-reacting material negative (CRM-), the plasma FIX activity was <1% (Table 3). In a few cases, additional mRNA, immunoblot, and protein expression studies confirmed these findings: an insertion comprising 5 kb in exon 8 of *F9* has been reported in Airedale Terrier causing alternate splicing and abolishing of the FIX activity (<1%) [10]. Similarly, a complete absence of FIX activity due to a single missense mutation in exon 8 was found in the Cairn Terrier research colony [7]. Because no further protein investigations, such as mRNA, immunoblotting and protein expression studies, were pursued here, the reported residual plasma FIX activity of the index case and the presence of a truncated protein remains unknown.

Spontaneous hemarthrosis, hematoma formation without known inciting causes, and increased bleeding after trauma and surgery are well-recognized complications of severe hemophilia in humans, dogs, and other mammalian species, leading to major morbidity and mortality [1,3,4]. Similarly, all affected male purebred Newfoundland and hybrid dogs in the family reported here exhibited severe hemorrhage, including mediastinal and subcutaneous hematomas, hemarthrosis, and gingival bleeding following loss of deciduous teeth. In contrast, heterozygous females remained asymptomatic.

Current standard therapy of bleeding dogs with hemophilia B and those needing to undergo surgery includes *DEA 1* matched and crossmatched plasma transfusions, as successfully performed in the index case. After transfusions, the index case clinically improved and had shortening of the aPTT. Fresh whole blood may be administered when a hemophilic animal is also anemic or in situations where no plasma products are available. Fresh frozen plasma may be used for dogs with either hemophilia A or B, but due to the different sizes of FVIII and FIX proteins, cryo-poor plasma should be administered to dogs with hemophilia B, while cryoprecipitate should be used for hemophilia A. Canine cryo-poor plasma, which is rich in smaller proteins such as FIX, is considerably less expensive than fresh frozen plasma and is preferred for hemophilia B patients, when available [3,30,31].

Hemophiliac patients arise sporadically from *de novo* mutations in maternal oocytes. Occasionally a variant is passed on over generations via the maternal genomic DNA, for example, the male descendants of Queen Victoria with hemophilia B in the royal families [32]. Similarly, the occurrence of the same 1.5 kb insertion *F9* variant was reported in few families of German Wirehaired Pointers from the USA and Europe [11]. To a lesser extent, familial occurrences of hemophilia B have been described in several Rhodesian Ridgebacks in Germany [12].

The single base insertion segregated completely in the described family of Newfoundland and Newfoundland-Standard Poodle hybrid dogs, with all tested males being hemizygous for the variant showing serious bleeding tendencies. Pedigree analysis and genotyping revealed that the pathogenic variant must have originated in the granddam with two female offspring (heterozygous for variant and thus, carriers) producing affected males or her maternal ancestors, which were purebred Newfoundland dogs; they either passed away or were not available for testing. Thereby, the pathogenic variant in the index case and related dogs was not de novo but passed on maternally to the offspring in this family.

Additionally, the variant described here was not found among 582 canine whole genome sequences deposited in DBVDC [18], including two Newfoundland dogs, one Landseer dog and two Standard Poodles. Moreover, genotyping of 75 Newfoundland dogs and Poodles for the insertion did not find any dogs with the insertion. According to the Comparative Coagulation Laboratory at Cornell University in Ithaca, a national referral center for diagnostics of bleeding disorders in animals, no hemophiliac Newfoundland dogs have been previously found (M.B. Brooks, personal communication, 2021). Thus, these results suggest that the *F9* insertion in Newfoundland dogs occurred recently and is restricted to a small family rather than widespread within the breed. 

While heterozygous females are clinically asymptomatic, a pathogenic *F9* variant may be inferred from pedigree analyses and plasma FIX activity may be half-normal, the best approach is genotyping for the specific pathogenic *F9* variant. In this study, the Newfoundland dam of the bleeding index case, was initially genotyped for known published *F9* variants by two commercial canine DNA panel testing laboratories and was found to be wildtype. This is not surprising as those published and tested for *F9* variants are breed specific and/or even isolated to a single case or family (Table 3). Thus, these non-breed specific results can be misleading breeders, pet owners, and attending veterinary clinicians. Indeed, when genotyping the dam and other family members for the newly identified insertion in the index case, the dam as well as the tested sister were found to be carriers. The identified carrier females and their common dam and granddam were spayed or deceased, respectively, and are no longer a threat to spreading this pathogenic variant. Although there are other untested related dogs in this family, it seems unlikely that this pathogenic *F9* variant is widespread. Nevertheless, it was advised that any related Newfoundland dog to this family with bleeding or intended for breeding should be genotyped. Any male with the pathogenic *F9* insertion variant should be carefully managed due to increased risk for bleeding or likely humanely euthanized because of poor prognosis, whereas any female testing heterozygous for the specific *F9* variant does not exhibit an increased bleeding tendency, but should be spayed (no excessive bleeding expected when heterozygous) and excluded from breeding (half of males affected).

## 5. Conclusions

Factor IX deficiency, known as hemophilia B, is an X-linked recessive hereditary coagulopathy with severe bleeding tendency and has been reported in various species including multiple canine breeds, but not previously in Newfoundland dogs. It should be noted that DNA variants are typically breed specific and can be caused by *de novo* mutations in any dog and family. The extent of the spread of the pathogenic variant in the Newfoundland breed is currently unknown, but the *F9* insertion seems to be limited to this family.

## Figures and Tables

**Table 1:** Routine hemostasis test results for a bleeding male Newfoundland–Parti Standard Poodle puppy with hemophilia B: Case #1.

Day	Time of Day	PCV (%)/ TP (g/dL)	aPTT/ PT (s) *	Comments
1	17	30/ND	240/11	Admission, dyspneic
2	08	13/5.0	>300/11	Progressive dyspnea, ventilated
	10	ND/ND	162/ND	Post pRBC transfusions **
	14	18/5.0	133/ND	Post thoracic surgery and FFP transfusion
	23	ND/ND	135/ND	Post additional FFP transfusion
3	07	20/6.0	ND/ND	Clinically stable
4	14	24/5.0	126/11	Clinically stable
5	10	35/7.0	124/ND	Prior to FFP transfusion **
	22	34/8.0	129/ND	Post FFP transfusion
6	10	ND/ND	105/ND	Post FFP transfusion
10	08	41/8.0	>300/ND	Severe chest wall hematoma
11	10	20/6.0	ND/ND	Euthanasia
Reference Ranges		30-45/5.5-7.2	72-102/11-17	

* Coag Dx^TM^ Analyzer (IDEXX, Portland, ME, USA) with citrated fresh whole blood. ** prior to and post refer to time measured related to nearby transfusion. Dog was discharged from clinic between Day 7 to Day 9. PCV, packed cell volume; TP, total protein; aPTT, activated partial thromboplastin time; PT, prothrombin time; ND, not determined; pRBC, packed red blood cells; FFP, fresh frozen plasma. Abnormal values are shown bold.

**Table 2:** Plasma hemostasis panel test results for a bleeding male Newfoundland–Parti Standard Poodle hybrid puppy with hemophilia B: Case #1.

Hemostatic Parameters	Unit	Results		Reference Range
		Day 2	Day 10	
PT	s	11.9	ND	11.0–15.5
aPTT	s	25.0	ND	8.5–15.5
TCT	s	4.5	ND	5.0–9.0
FVII	%	123	100	50–150
FVIII	%	104	51	50–200
FIX	%	11	3	50–150
FX	%	166	106	80–175
FXI	%	77	56	60–150
FXII	%	84	64	60–150
vWF	%	141	ND	70–180
FIX Inhibitor	BU/mL	ND	0.15	0–1.00

Performed with citrated plasma at Comparative Coagulation Laboratory, Cornell University, Ithaca, NY, USA. PT, prothrombin time; aPTT, activated partial thromboplastin time; TCT, thrombin clotting time; vWF, von Willebrand factor; %, activity of factor in percent of pooled plasma from healthy dogs; BU/mL, Bethesda unit per mL (a BU titer of <0.5 is considered not inhibiting activity of endogenous or transfused coagulation factors); ND, not determined. Abnormal values are shown bold.

**Table 3:** Characteristics of hemophilia B in dogs with known *F9* variants associated with hemophilia B in dogs.

Breed ^a^	Position in Genome	cDNA & Amino Acid Position	Type of Mutation	FIX Activity (%) ^b^	aPTT (s) ^c^	Clinical Signs	Occurrence
Cairn Terrier [7,22]	g.109532018G>A Exon 8	c.1253G>A p.Gly418Glu *	missense	0	70–90	Hemarthrosis, hematoma, CNS bleeding, mediastinal & cavity bleeding	Research Colony
Lhasa Apso [8]	g.109521356_109521361delinsT Intron 5	c.548_553delinsT p.Arg183LeufsTer3	deletion, insertion	<1	48/60	Hemorrhage, not specified	Single cases
Labrador Retriever [9]	complete deletion of factor IX gene	-	deletion	1	>60	Hematoma, hemarthrosis, prolonged bleeding from minor wounds	Family
Pit Bull Terrier [10]	deletion of entire 5´region extending to exon 6	-	deletion	<1	86–120	Hemarthrosis, intramuscular hematoma, epistaxis	Single case
Airedale Terrier [10]	g.109532012_13ins(5 kb) Exon 8	c.1247_1248ins(5kb)	insertion	<1	86–120	Hemarthrosis, intramuscular hematoma, epistaxis	Single case
German Wirehaired Pointer [11]	g.109521130_109521131insLINE1 Intron 5	-	insertion	2.4–6.4	Prolonged ^d^	Hematoma, hemarthrosis, excessive bleeding after surgery & minor wounds	several cases in Northern America & Europe
Rhodesian Ridgeback [12]	g.109530847G>A Exon 7	c.731G>A p.Gly237Glu	missense	∼1	Prolonged ^d^	Spontaneous hemorrhage & severe bleeding after minor surgeries	Family
Hovawart [13]	g.109501492delC Promoter	-	missense	2–5	3–5 fold increase	Umbilical & gingival bleeding, hemarthrosis, hematomas & hemorrhage after surgery & vaccination	Family
Newfoundland & Standard Poodle hybrid dogs *	g.109531586_109531587insA Exon 8	c.821_822insA p.Asn274LysfsTer23	insertion	11/3	25 ^e^	Hemarthrosis, hematoma, hemorrhage when losing deciduous teeth & after vaccination	Family

^a^ Listed in order of year published; ^c^ Normal upper limit of aPTT is ~25.0 s; ^d^ No specific times given; ^e^ Normal upper limit of aPTT is 15.5 s, several other aPTT measurements with inhouse instruments were also markedly prolonged (Table 1). ^b^ FIX activity, compared to pooled canine sample plasma assigned 100%; aPTT, normal ranges varied between 8-30 s; Updated variant nomenclature according to Human Genome Variation Society (HGVS) Recommendations for the Description of Sequence Variants [23]. Reference canine *F9* gene sequence (CanFam3.1, NC_006621.3, ChrX:109,501,341 to 109,533,798), reference canine *F9* amino acid sequence (NP_001003323.1) and reference canine *F9* cDNA sequence (NM_001003323.2) [15]. * Indicates family of dogs studied in this report. Canine hemophilia B cases without molecular genetic studies are not included.

## Data Availability

Not applicable.

## Supplementary Materials

The following are available online at https://www.mdpi.com/article/10.3390/genes12101491/s1, Table S1: Primer sequences used for DNA amplification and sequencing of canine *F9* exons and adjacent regions and primer sequences used for TaqMan® SNP Genotyping Assay for *F9* insertion, Figure S1: Amino acid sequence alignments of index case, reference canine, human, and feline FIX protein regions.
