# Supplementary material for: A Single Base Insertion in F9 Causing Hemophilia B in a Family of Newfoundland–Parti Standard Poodle Hybrid Dogs
Source: Genes (Basel). 2021 Sep 24;12(10):1491. doi: 10.3390/genes12101491 (PMC8535623; doi:10.3390/genes12101491)
Supplement: Supplementary file 1 [file genes-12-01491-s001.zip › genes-1385087-supplementary.pdf]

Supplements to Manuscript:

A single base insertion in *F9* causing hemophilia B in a family of Newfoundland-Parti Standard Poodle hybrid dogs.

Supplemental Table S1: Primer sequences used for DNA amplification and sequencing of canine *F9* exons and adjacent regions and primer sequences used for TaqMan® SNP Genotyping Assay for *F9* insertion.

Exon	Binding Site	Primer Sequence	Amplicon Size (bp)
1	5' UTR	F: 5'-TGTGTCACTTCCGGCTTCAG-3'	67
	Intron 1	R: 5'-TCTACAGCTAGAAGACAAGCATAC-3'	
2	Intron 1	F: 5′-TTGAATTTCAGTTTTTCTTGATCG-3′	164
	Intron 2	R: 5′-TGCATCTGAAGAACATTACGC-3′	
3	Intron 2	F: 5'-GCAGTTTTGAAGAAGCACGGG-3'	25
	Intron 3	R: 5'-ACACAGAGAAAAGATACCTAATTCTCA-3'	
4	Intron 3	F: 5'-AAGACAGGGGCATCCCATAATC-3'	114
	Intron 4	R: 5'-CTTCCATTAAGTGTTCCTTACCAC-3'	
5	Intron 4	F: 5'-CTCCCAAGCCTCTTTCCATG-3'	129
	Intron 5	R: 5'-CAAACAGGGTTTATGAAAGTATGTGAAC-3'	
6	Intron 5	F: 5'-CGTTCCGCTTGCCAATGAAAAATA-3'	197
	Intron 6	R: 5'-CTCTGGGCTCCAGTTTTGAC-3'	
7	Intron 6	F: 5'-ACACCCCTGCCTATCAACAG-3'	115
	Intron 7	R: 5'-GCTCCTCTAGCATTAGCCC-3'	
8	Intron 7	F: 5'-CCTTGGCAAATACGTTTATGTGTAAG-3'	548
	3' UTR	R: 5'-TGCCAAGGAGGGGAGAATC-3'	
TaqMan® SNP Genotyping Assay			
8	Intron 7	F: 5'-TGTAAGTCTACCTGTGACTGAAGAGAA-3'	90
	3' UTR	R: 5'-CGTTTCGCTTCTGCTCTGTATGT-3'	
	VICTM	5'-TCTTCTCGGTGTTATGC-3'	
	FAMTM	5'-TTCTCGGTGTTTATGC-3'	

bp, base pairs; F, forward primer; R, reverse Primer; UTR, untranslated region; VIC™, Dye label at 5' end; FAM™, Dye label at 5' end (Thermo Fisher Scientific)

Standard PCR protocol was used: 1) Pre- Denaturation (95°C, 5 min); 2) Denaturation (95°C, 0.5 min); 3) Annealing (59.5°C, 0.5 min); 4) Elongation (72°C, 1 min); 5) Denaturation 95°C, 45 sec); 6) Annealing (59.5°C, 45 sec); 7) Elongation (72°C, 45 sec); 8) Final elongation (72°C, 10 min); and 9) Cooling (4°C, ~2 hours). Step 2-4 (9 cycles), and step 5-7 (30 cycles).

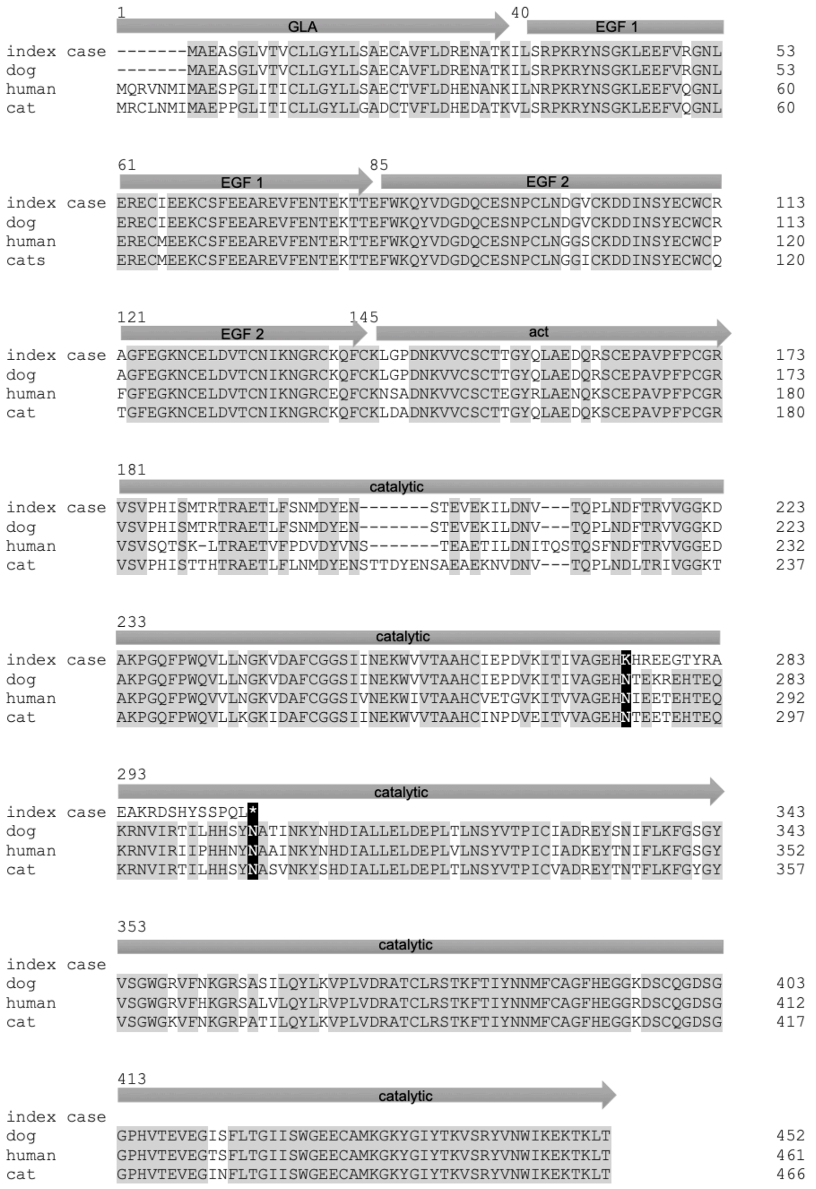

Supplemental Figure S1. Amino acid sequence alignments of index case, reference canine, human, and feline FIX protein regions.
The FIX amino acid sequences were aligned using the multiple sequence alignment tool Clustal Omega [25].
Residue numbers are labeled according to the human FIX sequence. The conserved amino acids between index case, dog, human, and cat are shaded in grey. The amino acid at the position of the variant in the hemophilic hybrid dog (index case), which is causing a frameshift, is shaded black and the first premature stop codon is depicted by an asterisk.
Domains are indicated by solid lines and numbers above the alignment, according to structure and function of human FIX [23]. Arrows above sequences display the different domains numbered according to the human FIX protein regions. GLA, gamma-carboxyglutamic acid-rich domain; EGF1, epidermal growth factor like domain 1; EGF2, epidermal growth factor like domain 2; act, aspartate kinase-chorismite mutase.
NCBI database accession numbers are: NP_000124.1 (human), NP_001009377.1 (feline) NP_001003323.1 (canine).
